# Feasibility of anthropometric indices to identify dyslipidemia among adults in Jilin Province: a cross-sectional study

**DOI:** 10.1186/s12944-017-0648-6

**Published:** 2018-01-22

**Authors:** Kaixin Zhang, Qian Zhao, Yong Li, Qing Zhen, Yaqin Yu, Yuchun Tao, Yi Cheng, Yawen Liu

**Affiliations:** 1 Department of Epidemiology and Biostatistics, School of Public Health, Jilin University, Changchun, 130021 China; 2 The Cardiovascular Center, The First Hospital of Jilin University, Changchun, 130021 China

**Keywords:** Dyslipidemia, Anthropometric indices, Serum lipid indices

## Abstract

**Background:**

Dyslipidemia and other cardiovascular disease (CVD) risk factors have a strong association with obesity. Anthropometric indices have been widely used to evaluate obesity in clinical and epidemiological studies. We aim to investigate association between serum lipid levels and different anthropometric indices.

**Methods:**

Our study included 17,554 participants. We mainly investigated area under the receiver operating characteristic (AUROC) curves and optimal operating points (OOPs) between the anthropometric indices and serum lipid levels or categories of abnormal serum lipid indices.

**Results:**

For predicting one/two categories of abnormal serum lipid indices among the anthropometric indices, AUROC value of WC was the highest in men (0.718), and AUROC values of BRI and WHtR were the highest in women (0.700 and 0.700) (all *P* < 0.001); OOP of WC was 82.450 in men; OOPs of BRI and WHtR were 3.435 and 0.504 in women. For predicting three/more categories of abnormal serum lipid indices among the anthropometric indices, AUROC value of WC was the highest in men (0.806), and AUROC values of BRI and WHtR were the highest in women (0.783 and 0.783) (all *P* < 0.001); OOP of WC was 84.150 in men; OOPs of BRI and WHtR were 3.926 and 0.529 in women.

**Conclusions:**

WC was a good predictor for one/two or three/more categories of abnormal serum lipid indices in men. However, BRI and WHtR were good predictors for one/two or three/more categories of abnormal serum lipid indices in women. ABSI showed the weakest predictive power.

**Electronic supplementary material:**

The online version of this article (10.1186/s12944-017-0648-6) contains supplementary material, which is available to authorized users.

## Background

Dyslipidemia is a critical risk-factor of cardiovascular disease (CVD) [1], contributing to mortality in patients with CVD [2, 3]. Dyslipidemia is defined by abnormal levels of total cholesterol (TC), or low-density cholesterol (LDL-C), or high-density cholesterol (HDL-C), or triglycerides (TGs) individually or in combination, which are based on biochemical measurement [1]. Although limited cost is required for the measurement of TC, TG, LDL-C, and HDL-C for one person, financial consumption on the basis of large population has brought huge socio-economic burden to a nation. For this reason, dyslipidemia costs $16 billion every year in China [4]. Screening population at high risk of dyslipidemia, using a simple, convenient, and cost-effective way, rather than measuring TG, TC, LDL-C, and HDL-C, is necessary for controlling CVD.

Dyslipidemia and other CVD risk factors (hypertension and diabetes) have a strong association with obesity [5]. Anthropometric indices have been widely used to evaluate obesity in clinical and epidemiological studies [6], because such measurements only require height weight scales and tapes that are robust and inexpensive.

Body mass index (BMI) reflects the overall distribution of body fat [7], which is a widely accepted anthropometric index of overweight and obesity [8]. Risk of CVD and/or Metabolic syndrome (MetS) increases with increasing of BMI [9]. However, compared with BMI, waist-to-hip ratio (WHR) and waist circumference (WC) can better reflect the accumulation of intra-abdominal fat [10–12]. WHR has also been proposed as a predictor of CVD risk factors among Tehranian adult men [12]. WHR has the weakest association with CVD risks among WC, BMI, and waist-to-height ratio (WHtR) [13], largely because nonobese individuals theoretically have the same WHR as obese ones in that WHR can remain constant with changes in weight [14]. Notably, WC better describes abdominal shape than BMI or WHR, and is highly associated with CVD risk factors, especially diabetes [15–17].

WHtR has been proposed as an indicator of abdominal obesity to evaluate variations of body size [18–20]. Several studies on Asian and Caucasian populations have found WHtR is superior to WC in identifying cases with CVD risk factors [12, 18, 21–24]. Furthermore, meta-analyses support those findings [25, 26].

New anthropometric indices, such as a body shape index (ABSI) and body roundness index (BRI) has been proposed recently. ABSI has been found to be more correlated with mortality rate than BMI or WC [27–29]. BRI predicts the percentage of body fat, evaluating health status. Up to date, only a few studies have investigated whether ABSI and BRI are suitable predictors for identifying CVD and/or MetS risk factors [30, 31].

It is still unclear which anthropometric index (BMI, WC, WHR, WHtR, ABSI, and BRI) could be the most appropriate predictor for identifying dyslipidemia in northeast China. In this study, we investigated association between serum lipid levels and the anthropometric indices in participants from Jilin Province to explore predictive ability of those indices for dyslipidemia.

## Methods

### Study population

**Fig. 1:**
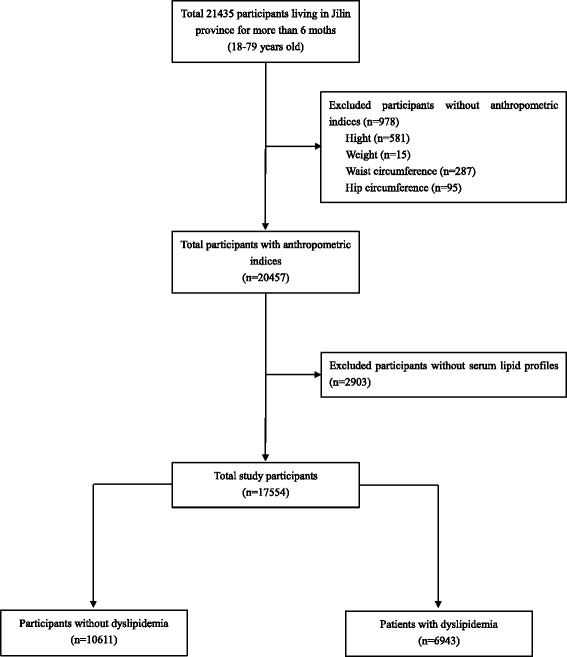
Flow chart for this investigation

The data in this study were collected from a cross-sectional survey of chronic diseases and related risk factors among adults in Jilin Province, China in 2012. The survey used a multistage cluster random sampling design to select a representative sample population aged from 18 to 79 years old who had lived in Jilin Province for more than 6 months in nine different cities/prefecture in Jilin Province, including Changchun, Jilin, Siping, Liaoyuan, Tonghua, Baishan, Songyuan, Baicheng, and Yanbian Korean Autonomous Prefecture. The survey involved 21,435 participants. Questionnaires, data of anthropometric measurements, and data of fasting blood tests were collected from each participant. Demographic characteristics (sex, age, and nationality) were collected from the questionnaires. The anthropometric data (height, weight, WC, and hip circumference) were got using height weight scales and tapes. Fasting blood tests were performed after fasting at least 12 h. Finally, this study included 17,554 participants with complete sets of data (Fig. 1).

### Sampling method

We selected participants using multistage hierarchical random cluster sampling method. Firstly, according to the ratio of population, location, and level of economic development, we selected 32 representative districts/counties from the nine cities/prefecture of Jilin Province. Secondly, we randomly selected three or four towns from each of the selected district/county using probability proportionate to size sampling. Thirdly, we randomly selected three administrative villages from each of the selected town using probability proportionate to size sampling. Fourthly, we selected one sub-village from each of the selected administrative village using simple random sampling. Finally, we randomly selected an adult aged 18 to 79 years old from every household in the each selected sub-village.

### Calculation of anthropometric indices

WHR, WHtR [7, 32], BMI [33], ABSI [27], and BRI [28] were calculated using the following formulas.

$$ \mathrm{WHR}=\mathrm{WC}\ \left(\mathrm{cm}\right)/\mathrm{HC}\ \left(\mathrm{cm}\right) $$

$$ \mathrm{WHtR}=\mathrm{WC}\ \left(\mathrm{cm}\right)/\mathrm{height}\ \left(\mathrm{cm}\right) $$

$$ \mathrm{BMI}=\mathrm{Weight}\ \left(\mathrm{kg}\right)/\mathrm{Height}\ \left({\mathrm{m}}^2\right) $$

$$ \mathrm{ABSI}=\frac{\mathrm{wc}}{BMI^{\raisebox{1ex}{$2$}\!\left/ \!\raisebox{-1ex}{$3$}\right.}{height}^{1/2}} $$

$$ BRI=364.2-365.5\times \sqrt{1-\left(\frac{{\left( wc/\left(2\pi \right)\right)}^2}{{\left(0.5 height\right)}^2}\right)} $$

### Laboratory assay

Fasting serum samples were used to measure TG, TC, LDL-C, and HDL-C using MODULE P800 biochemical analysis machine (Roche Co., Ltd. Shanghai, China).

Dyslipidemia was defined according to the Chinese guidelines on the prevention and treatment of dyslipidemia in adults (2007) [34]: TG ≥ 2.26 mmol/L (200 mg/dL) as high; TC ≥ 6.22 mmol/L (240 mg/dL) as high; LDL-C ≥ 4.14 mmol/L (160 mg/dL) as high; and HDL-C < 1.04 mmol/L (40 mg/dL) as low.

### Statistical analysis

All statistical analyses were performed using SPSS version 21.0 (SPSS Inc., Chicago, IL, USA). Mean and standard deviation were used to express normally distributed continuous variables, and median and Q1 to Q3 were used to express abnormally distributed continuous variables, with Q1 being the 25th percentile and Q3 being the 75th percentile. Continuous variables were tested for normality using the Kolmogorov-Smirnov test. Comparisons between men and women were performed using two independent samples t-test or Mann-Whitney U test for continuous variables. Relationship between the anthropometric indices and serum lipid levels was analyzed using Spearman’s correlation analysis and Partial correlation analysis. Receiver operating characteristic  (ROC) analysis was used to calculate the area under ROC curves (AUROC) between the anthropometric indices and serum lipid levels. Considering that the optimal cut-off point balance sensitivity (SEN) and specificity (SPE) of anthropometric index, we used optimal operating point (OOP) which is the maximum Youden index (SEN + SPE −1) [35] to select the optimal cut-off point to predict dyslipidemia. *P* < 0.05 was considered statistically significant.

## Results

### Baseline characteristics of the participants

Baseline characteristics of the participants stratified by gender are shown in Table 1. A total of 17,554 participants, including 10,611 normal people (60.448%) and 6943 patients with dyslipidemia (39.552%), were composed of 8080 men (46.029%) and 9474 women (53.971%). The median age of the participants was 48 years old in both men and women. For anthropometric indices, the medians of height, weight, hip circumference, WC, BMI, ABSI, and WHR were higher, but those of BRI and WHtR were lower in men (all *P* < 0.001). For serum lipid levels, the medians of TC level were similar (*P* = 0.121), LDL-C level (*P* < 0.001) and HDL-C level (*P* < 0.001) were higher, but TG level (*P* < 0.001) was lower in women.

**Table 1:** Descriptive baseline characteristics of the participants

	Men(8080)	Women(9474)	*t/Z*	*P*
Age (years)	48(37,57)	48(40,58)	-6.007	<0.001
Height (cm)	169.200(165.000,173.800)	157.500(153.500,161.300)	-93.614	<0.001
Weight (kg)	68.900(61.200,77.175)	59.200(53.100,65.900)	-54.837	<0.001
HC (cm)	95.200(90.600,100.000)	94.000(90.000,99.000)	-8.811	<0.001
WC (cm)	84.800(77.000,92.000)	80.000(73.000,87.200)	-26.242	<0.001
BMI (kg/m2)	24.152(21.719,26.680)	23.936(21.586,26.469)	-2.732	<0.001
ABSI (m11/6 kg-2/3)	0.078(0.075,0.081)	0.077(0.073,0.080)	-13.303	<0.001
BRI	3.385(2.594,4.187)	3.540(2.669,4.511)	-9.481	<0.001
WHR	0.885 ± 0.669	0.849 ± 0.073	34.087	<0.001
WHtR	0.501 ± 0.061	0.510(0.462,0.558)	-9.481	<0.001
TG(mmol/L)	1.570(1.040,2.540)	1.405(0.960,2.140)	-12.645	<0.001
TC(mmol/L)	4.770(4.160,5.460)	4.790(4.140,5.533)	-1.552	0.121
LDL-C(mmol/L)	2.830(2.310,3.398)	2.870(2.330,3.510)	-4.163	<0.001
HDL-C(mmol/L)	1.280(1.070,1.550)	1.380(1.160,1.640)	-16.211	<0.001

*HC* Hip Circumference, *WC* Waist Circumference, *BMI* Body Mass Index, *ABSI* A Body Shape Index, *BRI* Body Roundness Index, *WHR* Waist-to-Hip Ratio, *WHtR* Waist-to-Height Ratio, *TG* Triglyceride, *TC* Total Cholesterol, *LDL-C* Low Density Lipoprotein Cholesterol, *HDL-C* High Density Lipoprotein Cholesterol

### Relationship between anthropometric indices and serum lipid levels

We investigated the relationship between anthropometric indices and serum lipid levels using Spearman’s rank test (Table 2). For men, high correlations were identified between WC and TG level (*r* = 0.475, *P* < 0.001), between the levels of BRI and WHtR and TC level (both *r* = 0.270, *P* < 0.001), between the levels of BRI and WHtR and LDL-C level (both *r* = 0.227, *P* < 0.001), and between BMI and HDL-C level (*r* = −0.406, *P* < 0.001). For women, high correlations were identified between the levels of BRI and WHtR and TG level (both *r* = 0.469, *P* < 0.001), between the levels of BRI and WHtR and TC level (both *r* = 0.311, *P* < 0.001), between the levels of BRI and WHtR and LDL-C level (both *r* = 0.320, *P* < 0.001), and between WC and HDL-C level (*r* = −0.303, *P* < 0.001). After adjusting for age, for men, high correlations were identified between WHtR and the levels of TG, TC, and LDL-C (*r* = 0.326, 0.232, and 0.187, respectively; all *P* < 0.001), and between WC and HDL-C level (*r* = −0.376, *P* < 0.001). For women, high correlations were identified between WC and the levels of TG, TC, and LDL-C (*r* = 0.231, 0.131, and 0.154, respectively; all *P* < 0.001), and between WC and HDL-C level (*r* = −0.282, *P* < 0.001) (Table 3).

**Table 2:** Spearman correlation coefficient between anthropometric indices and serum lipid levels

	TG		TC		LDL-C		HDL-C	
	*r*	*P*	*r*	*P*	*r*	*P*	*r*	*P*
Total								
WC	0.471	<0.001	0.261	<0.001	0.248	<0.001	-0.360	<0.001
BMI	0.419	<0.001	0.218	<0.001	0.217	<0.001	-0.341	<0.001
ABSI	0.291	<0.001	0.231	<0.001	0.204	<0.001	-0.156	<0.001
BRI	0.458	<0.001	0.293	<0.001	0.281	<0.001	-0.316	<0.001
WHR	0.452	<0.001	0.269	<0.001	0.233	<0.001	-0.306	<0.001
WHtR	0.458	<0.001	0.293	<0.001	0.281	<0.001	-0.316	<0.001
Men								
WC	0.475	<0.001	0.249	<0.001	0.213	<0.001	-0.399	<0.001
BMI	0.461	<0.001	0.224	<0.001	0.201	<0.001	-0.406	<0.001
ABSI	0.231	<0.001	0.187	<0.001	0.139	<0.001	-0.140	<0.001
BRI	0.468	<0.001	0.270	<0.001	0.227	<0.001	-0.375	<0.001
WHR	0.443	<0.001	0.261	<0.001	0.197	<0.001	-0.316	<0.001
WHtR	0.468	<0.001	0.270	<0.001	0.227	<0.001	-0.375	<0.001
Women								
WC	0.455	<0.001	0.286	<0.001	0.300	<0.001	-0.303	<0.001
BMI	0.383	<0.001	0.214	<0.001	0.232	<0.001	-0.283	<0.001
ABSI	0.329	<0.001	0.267	<0.001	0.260	<0.001	-0.152	<0.001
BRI	0.469	<0.001	0.311	<0.001	0.320	<0.001	-0.291	<0.001
WHR	0.450	<0.001	0.299	<0.001	0.295	<0.001	-0.270	<0.001
WHtR	0.469	<0.001	0.311	<0.001	0.320	<0.001	-0.291	<0.001

*WC* Waist Circumference, *BMI* Body Mass Index, *ABSI* A Body Shape Index, *BRI* Body Roundness Index, *WHR* Waist-to-Hip Ratio, *WHtR* Waist-to-Height Ratio, *TG* Triglyceride, *TC* Total Cholesterol, *LDL-C* Low Density Lipoprotein Cholesterol, *HDL-C* High Density Lipoprotein Cholesterol

**Table 3:** Partial correlation coefficients between anthropometric indices and serum lipid levels^a^

	TG		TC		LDL-C		HDL-C	
	*r*	*P*	*r*	*P*	*r*	*P*	*r*	*P*
Total								
WC	0.308	<0.001	0.189	<0.001	0.175	<0.001	-0.347	<0.001
BMI	0.264	<0.001	0.175	<0.001	0.172	<0.001	-0.320	<0.001
ABSI	0.152	<0.001	0.096	<0.001	0.080	<0.001	-0.139	<0.001
BRI	0.269	<0.001	0.191	<0.001	0.184	<0.001	-0.305	<0.001
WHR	0.295	<0.001	0.168	<0.001	0.133	<0.001	-0.291	<0.001
WHtR	0.278	<0.001	0.197	<0.001	0.189	<0.001	-0.317	<0.001
Men								
WC	0.321	<0.001	0.221	<0.001	0.183	<0.001	-0.376	<0.001
BMI	0.303	<0.001	0.209	<0.001	0.180	<0.001	-0.367	<0.001
ABSI	0.153	<0.001	0.113	<0.001	0.074	<0.001	-0.141	<0.001
BRI	0.321	<0.001	0.226	<0.001	0.182	<0.001	-0.361	<0.001
WHR	0.308	<0.001	0.209	<0.001	0.149	<0.001	-0.305	<0.001
WHtR	0.326	<0.001	0.232	<0.001	0.187	<0.001	-0.367	<0.001
Women								
WC	0.231	<0.001	0.131	<0.001	0.154	<0.001	-0.282	<0.001
BMI	0.201	<0.001	0.121	<0.001	0.143	<0.001	-0.261	<0.001
ABSI	0.105	<0.001	0.052	<0.001	0.059	<0.001	-0.104	<0.001
BRI	0.215	<0.001	0.126	<0.001	0.146	<0.001	-0.259	<0.001
WHR	0.220	<0.001	0.115	<0.001	0.114	<0.001	-0.239	<0.001
WHtR	0.223	<0.001	0.130	<0.001	0.151	<0.001	-0.272	<0.001

*WC* Waist Circumference, *BMI* Body Mass Index, *ABSI* A Body Shape Index, *BRI* Body Roundness Index, *WHR* Waist-to-Hip Ratio, *WHtR* Waist-to-Height Ratio, *TG* Triglyceride, *TC* Total Cholesterol, *LDL-C* Low Density Lipoprotein Cholesterol, *HDL-C* High Density Lipoprotein Cholesterol

^a^Adjusted for age

### Relationship between anthropometric indices and categories of abnormal serum lipid indices

Because there are no reference and guideline to reveal relationship between anthropometric indices and categories of abnormal serum lipid indices, and there are few patients with three categories of abnormal serum lipid indices and four categories of abnormal serum lipid indices, we further classified the patients into two groups: patients with one/two categories of abnormal serum lipid indices (group 1) and patients with three/more categories of abnormal serum lipid indices (group 2). We investigated the relationship between anthropometric indices and categories of abnormal serum lipid indices using Spearman rank test (Table 4). For men, high correlation was identified between WC and categories of abnormal serum lipid indices (*r* = 0.383, *P* < 0.001). For women, high correlations were identified between the levels of BRI and WHtR and categories of abnormal serum lipid indices in the two groups (both *r* = 0.344, *P* < 0.001). After adjusting for age, for men, high correlation was identified between WC and categories of abnormal serum lipid indices (*r* = 0.376, *P* < 0.001). For women, high correlations were identified between the levels of WC, BMI, and WHtR and categories of abnormal serum lipid indices (all *r* = 0.239, *P* < 0.001) (Table 5).

**Table 4:** Spearman correlation coefficient between anthropometric indices and categories of abnormal serum lipid indices

	Categories of abnormal serum lipid indices	
	*r*	*P*
Total		
WC	0.363	<0.001
BMI	0.313	<0.001
ABSI	0.229	<0.001
BRI	0.351	<0.001
WHR	0.348	<0.001
WHtR	0.351	<0.001
Men		
WC	0.383	<0.001
BMI	0.366	<0.001
ABSI	0.186	<0.001
BRI	0.377	<0.001
WHR	0.347	<0.001
WHtR	0.377	<0.001
Women		
WC	0.333	<0.001
BMI	0.266	<0.001
ABSI	0.253	<0.001
BRI	0.344	<0.001
WHR	0.333	<0.001
WHtR	0.344	<0.001

*WC* Waist Circumference, *BMI* Body Mass Index, *ABSI* A Body Shape Index, *BRI* Body Roundness Index, *WHR* Waist-to-Hip Ratio, *WHtR* Waist-to-Height Ratio

**Table 5:** Partial correlation coefficients between anthropometric indices and categories of abnormal serum lipid indices^a^

	Categories of abnormal serum lipid indices	
	*r*	*P*
Total		
WC	0.331	<0.001
BMI	0.294	<0.001
ABSI	0.178	<0.001
BRI	0.314	<0.001
WHR	0.308	<0.001
WHtR	0.314	<0.001
Men		
WC	0.376	<0.001
BMI	0.360	<0.001
ABSI	0.189	<0.001
BRI	0.373	<0.001
WHR	0.345	<0.001
WHtR	0.373	<0.001
Women		
WC	0.239	<0.001
BMI	0.208	<0.001
ABSI	0.121	<0.001
BRI	0.239	<0.001
WHR	0.227	<0.001
WHtR	0.239	<0.001

*WC* Waist Circumference, *BMI* Body Mass Index, *ABSI* A Body Shape Index, *BRI* Body Roundness Index, *WHR* Waist-to-Hip Ratio, *WHtR* Waist-to-Height Ratio

^a^Adjusted for age

### AUROC and OOPs of anthropometric indices

We investigated AUROCs between dyslipidemia and the anthropometric indices to predict dyslipidemia. For men, AUROC value of WC was the highest among the anthropometric indices (AUROC = 0.726, *P* < 0.001). For women, AUROC values of BRI and WHtR were the highest among the anthropometric indices (both AUROC = 0.709, *P* < 0.001). Men had higher OOPs of WC, BMI, ABSI, and WHR (84.050, 23.817, 0.077, and 0.880 in men, or 79.250, 23.218, 0.076, and 0.847 in women). Women had higher OOPs of BRI and WHtR (3.435 and 0.504 in women, or 3.202 and 0.492 in men).

Next, we investigated AUROCs and OOPs between serum lipid levels and anthropometric indices. For men, AUROC value of WC was the highest for predicting TG abnormal level among the anthropometric indices (AUROC = 0.730, *P* < 0.001), and OOP of WC was 84.050; AUROC values of BRI and WHtR were the highest for predicting TC (both AUROC = 0.645, *P* < 0.001) or LDL-C abnormal levels (both AUROC = 0.633 , *P* < 0.001) among the anthropometric indices, OOP of BRI was 3.471 for TC or 3.204 for LDL-C, and OOP of WHtR was 0.508 for TC or 0.492 for LDL-C; AUROC value of BMI was the highest for predicting HDL-C abnormal level among the anthropometric indices (AUROC =0.695, *P* < 0.001), and OOP of BMI was 24.750. For women, AUROC values of BRI and WHtR were the highest for predicting TG (both AUROC = 0.715, *P* < 0.001), TC (both AUROC = 0.660, *P* < 0.001), or LDL-C abnormal levels (both AUROC = 0.662, *P* < 0.001) among the anthropometric indices, OOP of BRI was 3.436 for TG, 3.926 for TC, or 3.596 for LDL-C, and OOP of WHtR was 0.504 for TG, 0.512 for TC, or 0.512 for LDL-C; AUROC value of WC was the highest for predicting HDL-C abnormal level among the anthropometric indices (AUROC = 0.654, *P* < 0.001), and OOP of WC was 79.250 (Table 6, Additional file 1: Figure S1 A-H, Table 7).

**Table 6:** AUROCs for anthropometric indices and serum lipid levels

	AUROC(95%CI)			
	TG	TC	LDL-C	HDL-C
Men				
WC	0.730(0.719,0.742)*	0.636(0.616,0.655)*	0.621(0.599,0.642)*	0.694(0.681,0.708)*
BMI	0.727(0.716,0.739)*	0.626(0.607,0.645)*	0.607(0.585,0.629)*	0.695(0.682,0.708)*
ABSI	0.607(0.594,0.620)*	0.589(0.568,0.609)*	0.589(0.566,0.612)*	0.576(0.561,0.590)*
BRI	0.728(0.717,0.739)*	0.645(0.626,0.664)*	0.633(0.611,0.654)*	0.683(0.669,0.696)*
WHR	0.718(0.706,0.729)*	0.631(0.611,0.650)*	0.612(0.590,0.634)*	0.661(0.647,0.674)*
WHtR	0.728(0.717,0.739)*	0.645(0.626,0.664)*	0.633(0.611,0.654)*	0.683(0.669,0.696)*
Women				
WC	0.711(0.700,0.723)*	0.645(0.629,0.661)*	0.650(0.634,0.666)*	0.654(0.638,0.669)*
BMI	0.672(0.660,0.684)*	0.601(0.583,0.618)*	0.613(0.596,0.631)*	0.627(0.612,0.643)*
ABSI	0.656(0.643,0.668)*	0.649(0.633,0.666)*	0.635(0.617,0.652)*	0.597(0.581,0.613)*
BRI	0.715(0.703,0.726)*	0.660(0.643,0.676)*	0.662(0.646,0.678)*	0.646(0.631,0.662)*
WHR	0.714(0.703,0.726)*	0.658(0.642,0.675)*	0.648(0.632,0.665)*	0.646(0.630,0.661)*
WHtR	0.715(0.703,0.726)*	0.660(0.643,0.676)*	0.662(0.646,0.678)*	0.646(0.631,0.662)*

*WC* Waist Circumference, *BMI* Body Mass Index, *ABSI* A Body Shape Index, *BRI* Body Roundness Index, *WHR* Waist-to-Hip Ratio, *WHtR* Waist-to-Height Ratio, *TG* Triglyceride, *TC* Total Cholesterol, *LDL-C* Low Density Lipoprotein Cholesterol, *HDL-C* High Density Lipoprotein Cholesterol

*P < 0.001

**Table 7:** Optimal operating points of anthropometric indices for predicting abnormal serum lipid levels

	TG			TC			LDL-C			HDL-C		
	OOP	SEN(%)	SPE(%)	OOP	SEN(%)	SPE(%)	OOP	SEN(%)	SPE(%)	OOP	SEN(%)	SPE(%)
Men												
WC	84.050	76.015	60.097	84.350	69.829	51.318	80.750	80.789	37.242	85.350	70.412	59.265
BMI	24.512	70.269	65.015	23.817	71.805	48.436	23.543	72.213	44.805	24.750	67.024	63.291
ABSI	0.077	69.225	47.612	0.077	69.170	45.581	0.078	57.804	55.556	0.077	69.283	43.383
BRI	3.253	78.224	57.324	3.471	67.721	55.061	3.204	75.129	46.285	3.301	73.574	53.527
WHR	0.881	76.215	56.967	0.897	62.187	57.861	0.863	79.760	37.762	0.890	67.871	57.458
WHtR	0.492	79.911	55.607	0.508	66.930	55.785	0.492	75.129	46.259	0.497	73.518	53.574
Women												
WC	81.250	70.699	62.335	80.450	67.262	54.108	79.550	72.527	50.325	79.250	73.697	50.590
BMI	22.987	81.106	45.580	23.327	70.273	44.977	23.500	70.030	47.114	24.363	63.192	57.032
ABSI	0.077	69.808	54.924	0.077	65.193	57.674	0.076	71.029	49.380	0.076	64.475	50.833
BRI	3.436	78.856	54.597	3.926	59.831	53.953	3.596	69.930	54.196	3.435	72.975	50.067
WHR	0.862	66.526	65.059	0.829	80.903	42.088	0.831	79.520	42.925	0.861	60.946	60.423
WHtR	0.504	78.762	54.652	0.512	69.332	53.953	0.512	70.230	53.889	0.504	72.975	50.067

*WC* Waist Circumference, *BMI* Body Mass Index, *ABSI* A Body Shape Index, *BRI* Body Roundness Index, *WHR* Waist-to-Hip Ratio, *WHtR* Waist-to-Height Ratio, *TG* Triglyceride, *TC* Total Cholesterol, *LDL-C* Low Density Lipoprotein Cholesterol, *HDL-C* High Density Lipoprotein Cholesterol, *OOP* Optimal Operating Points, *SEN* Sensitivity; SPE: Specificity

We further investigated AUROCs and OOPs between categories of abnormal serum lipid indices and anthropometric indices. For men, AUROC values of WC was the highest for predicting categories of abnormal serum lipid indices in group 1 (AUROC = 0.718, *P* < 0.001) or in group 2 (AUROC = 0.806, *P* < 0.001) among the anthropometric indices, OOP of WC was 82.450 for group 1 or 84.150 for group 2. For women, AUROC values of BRI and WHtR were the highest for predicting categories of abnormal serum lipid indices in group 1 (both AUROC = 0.700, *P* < 0.001) or in group 2 (both AUROC = 0.783, *P* < 0.001) among the anthropometric indices, OOP of BRI was 3.435 for group 1 or 3.926 for group 2, and OOP of WHtR was 0.504 for group 1 or 0.529 for group 2 (Table 8, Additional file 1: Figure S1 I-L, Table 9).

**Table 8:** AUROCs for anthropometric indices and categories of abnormal serum lipid indices

	AUROC(95%CI)	
	One/two	Three/more
Men		
WC	0.718(0.707,0.730)*	0.806(0.785,0.826)*
BMI	0.712(0.701,0.724)*	0.793(0.770,0.815)*
ABSI	0.603(0.591,0.616)*	0.663(0.635,0.690)*
BRI	0.714(0.703,0.726)*	0.805(0.784,0.826)*
WHR	0.702(0.691,0.714)*	0.768(0.745,0.792)*
WHtR	0.714(0.703,0.726)*	0.805(0.784,0.826)*
Women		
WC	0.697(0.686,0.708)*	0.773(0.752,0.794)*
BMI	0.660(0.648,0.671)*	0.713(0.688,0.737)*
ABSI	0.648(0.637,0.660)*	0.721(0.696,0.746)*
BRI	0.700(0.689,0.711)*	0.783(0.762,0.804)*
WHR	0.696(0.685,0.708)*	0.774(0.752,0.796)*
WHtR	0.700(0.689,0.711)*	0.783(0.762,0.804)*

*WC* Waist Circumference, *BMI* Body Mass Index, *ABSI* A Body Shape Index, *BRI* Body Roundness Index, *WHR* Waist-to-Hip Ratio, *WHtR* Waist-to-Height Ratio

*P < 0.001

**Table 9:** Optimal operating points of anthropometric indices for predicting categories of abnormal serum lipid indices

	One/two			Three/more		
	OOP	SEN(%)	SPE(%)	OOP	SEN(%)	SPE(%)
Men						
WC	82.450	75.462	57.737	84.150	84.640	64.224
BMI	23.877	70.129	61.789	24.787	77.116	71.441
ABSI	0.077	66.523	50.055	0.076	82.759	42.993
BRI	3.202	73.274	59.752	3.516	80.878	68.408
WHR	0.880	71.270	59.863	0.897	73.041	69.648
WHtR	0.492	73.274	59.752	0.508	80.878	68.408
Women						
WC	79.250	71.438	58.582	80.750	80.217	63.243
BMI	23.218	73.863	51.313	24.216	70.732	61.913
ABSI	0.076	70.309	52.855	0.077	73.984	59.928
BRI	3.435	72.434	58.500	3.926	72.629	71.529
WHR	0.842	71.073	57.434	0.863	72.358	68.608
WHtR	0.504	72.434	58.500	0.529	72.629	71.529

*WC* Waist Circumference, *BMI* Body Mass Index, *ABSI* A Body Shape Index, *BRI* Body Roundness Index, *WHR* Waist-to-Hip Ratio, *WHtR* Waist-to-Height Ratio, *OOP* Optimal Operating Points, *SEN* Sensitivity, *SPE* Specificity

## Discussion

This study mainly focusses on identifying the capacity of commonly used anthropometric indices (WC, BMI, ABSI, BRI, WHR, and WHtR) in prediction for dyslipidemia. Our results showed that all the anthropometric indices can predict dyslipidemia independently because they all had AUROCs >0.5.

Anthropometric indices (BMI, WC, WHR, WHtR, and ABSI) have positive correlation with TG, TC, and LDL-C levels, but have negative correlation with HDL-C level [36–39]. BRI positively correlates with TG and LDL-C levels, but negatively correlates with HDL-C level [40]. Our results also supported those previous results. Moreover, we identified that BRI also had positive correlation with TC levels.

Age-specific BMI and WC are associated with CVD risk factors, including fasting insulin, TG, HDL-C, and LDL-C among Chinese children [41]. A cohort study, which included 8940 Chinese adults, reveals that BMI is strongly associated with hypertension, and BMI has higher AUROC and prevalence ratio, while WC is associated with diabetes and dyslipidemia [42]. Age-specific WC is superior to BMI in predicting MetS in children [43]. Working Group on Obesity in China has also recommended that BMI is a better predictor of hypertension in adults, while WHtR and WC are more sensitive to predict diabetes and dyslipidemia [44].

In this study, we furthermore identified that WC, BMI, and WHtR were the best predictors for TG, HDL-C, and TC or LDL-C abnormal levels in men respectively; WHtR was the best predictor for TG, TC, or LDL-C abnormal levels, and WC was the best predictor for HDL-C abnormal level in women.

ABSI could be served as a substantial risk factor for premature mortality in Korean population [45]. However, ABSI has no evidence to distinguish between individuals with and without CVD or CVD risk factors, including dyslipidemia [46]. Compared with BMI and WC, ABSI has similar predictive ability for initial stage diabetes in a prospective cohort study in China which includes 687 people after a follow up of 15 years, indicating that ABSI in this respect is not superior to BMI or WC [47]. ABSI is not the best predictor of hypertension, diabetes, and dyslipidemia for Japanese adults in retrospective cohort of 48,953 Japanese adults during a follow-up of 4 years [30]. Our results supported those above results. Our results validated ABSI could not be used as the best sensitive predictor for abnormal TG, TC, LDL-C, and HDL-C levels respectively. In contrast, Haghighatdoost et al. have shown the highest odds ratio was observed for ABSI and MetS in different age and sex categories in a population-based cohort of 9555 Iranian adults aged ≥19 years [48]. The endpoint of variable choices [46] and the weak correlation between ABSI and height [48] may contribute to the discrepancy.

BRI has a good discriminative ability for either diabetes or CVD and its risk factors, and BRI has a larger AUROC value than BMI and WC [31, 46, 49]. BRI is superior to WHtR: BRI can accurately estimate the percentage of body fat and visceral adipose tissues [28]. In our study, BRI levels in men were different from those in women. For men, BRI was not the best predictor for dyslipidemia; however, for women, BRI was the best predictor for dyslipidemia. We found BRI could be used as the best sensitive predictor for TG abnormal level in women and TC or LDL-C abnormal level in men and women respectively.

To our knowledge, this is the first study to investigate relationship between the anthropometric indices and categories of abnormal serum lipid indices. Our results demonstrated WC could be used to identify all categories of abnormal serum lipid indices in men; WHtR and BRI could be used to identify all categories of abnormal serum lipid indices in women. Moreover, our results further revealed that the values of anthropometric indices (BMI, WC, WHR, WHtR, and BRI) increase with increasing categories of abnormal serum lipid indices in both men and women groups.

This study has limitations. First, pharmacological treatments, diets, and nutraceuticals can influence results. Second, ABSI was developed to predict mortality hazard in a follow-up study; however, we used ABSI to predict dyslipidemia in a cross-sectional study. Third, the sensitivity of BRI to determine risk of dyslipidemia in a clinical setting remains elusive.

## Conclusions

In our study, WC was a good predictor for one/two or three/more categories of abnormal serum lipid indices in men. However, BRI and WHtR were good predictors for one/two or three/more categories of abnormal serum lipid indices in women. ABSI showed the weakest predictive power. These indices are necessary for screening of dyslipidemia in clinical practice.

## Additional file

Additional file 1: Receiver Operating Characteristic (ROC) curves for anthropometric indices and serum lipid levels. (DOCX 334 kb)

## Abbreviations

ABSI: A Body shape index
AUROC: Area under the receiver operating characteristic curve
BMI: Body mass index
BRI: Body roundness index
CVD: Cardiovascular disease
HDL-C: High-density cholesterol
LDL-C: Low-density cholesterol
MetS: Metabolic syndrome
OOP: Optimal operating point
ROC: Receiver operating characteristic
SEN: Sensitivity
SPE: Specificity
TC: Total cholesterol
TGs: Triglycerides
WC: Waist circumference
WHR: Waist-to-hip ratio
WHtR: Waist-to-height ratio
