# Supplementary material for: Feasibility of anthropometric indices to identify dyslipidemia among adults in Jilin Province: a cross-sectional study
Source: Lipids Health Dis. 2018 Jan 22;17:16. doi: 10.1186/s12944-017-0648-6 (PMC5778621; doi:10.1186/s12944-017-0648-6)
Supplement: Additional file 1: — Receiver Operating Characteristic (ROC) curves for anthropometric indices and serum lipid levels. (DOCX 334 kb) [file 12944_2017_648_MOESM1_ESM.docx]

**Supplementary:**

A B


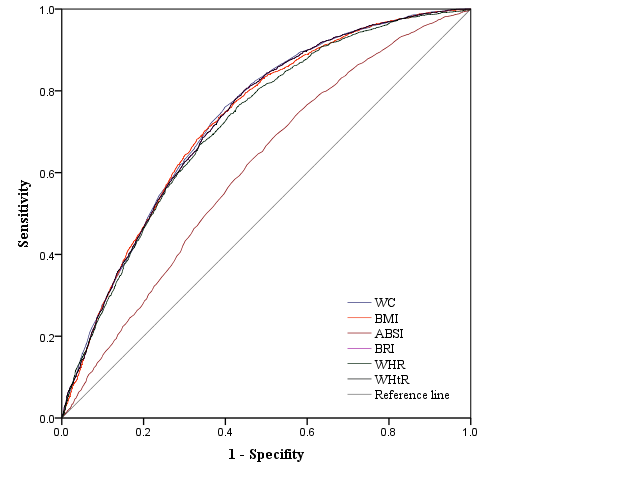

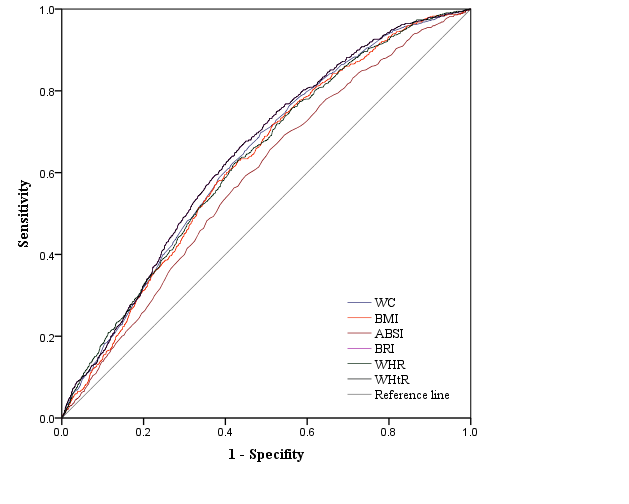
 C D


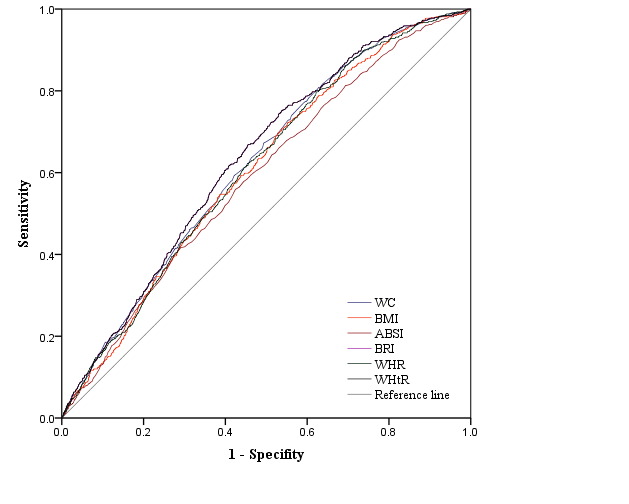

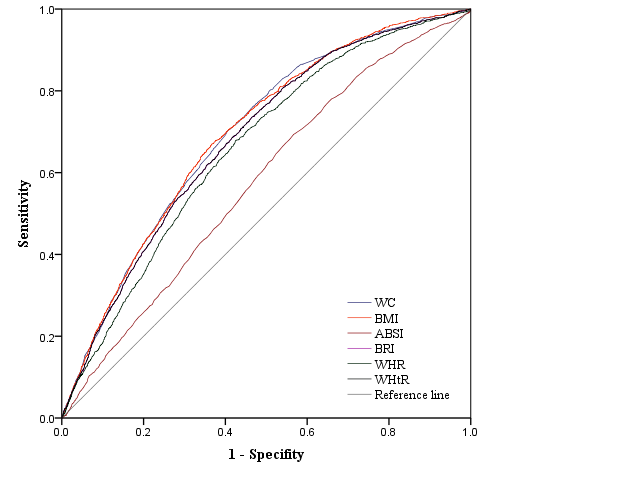


E F


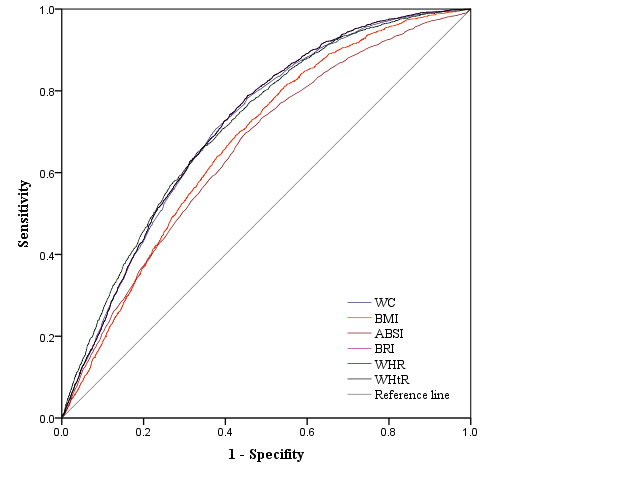

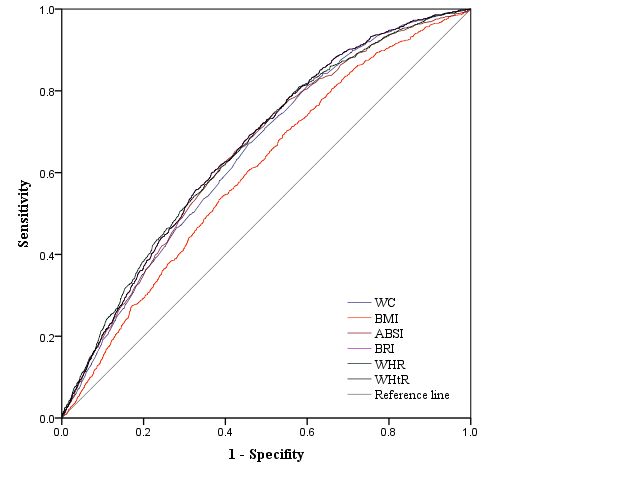


G H


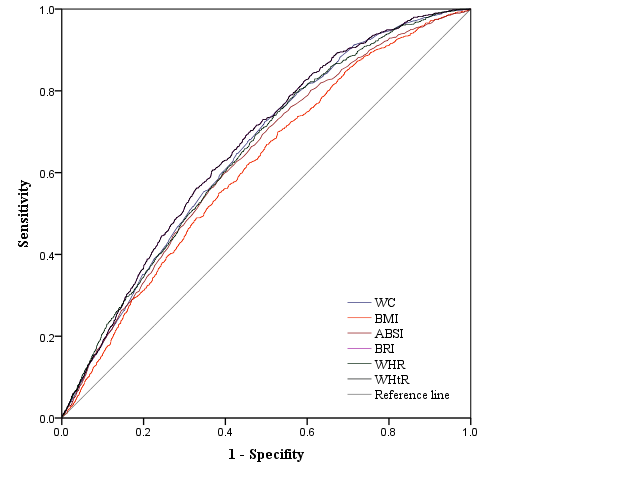

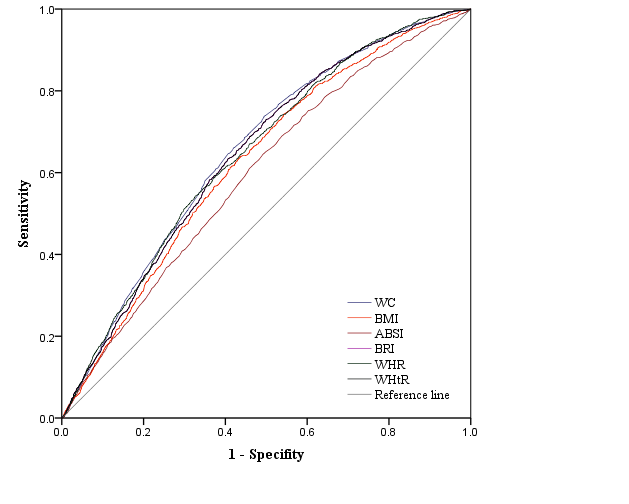


I J


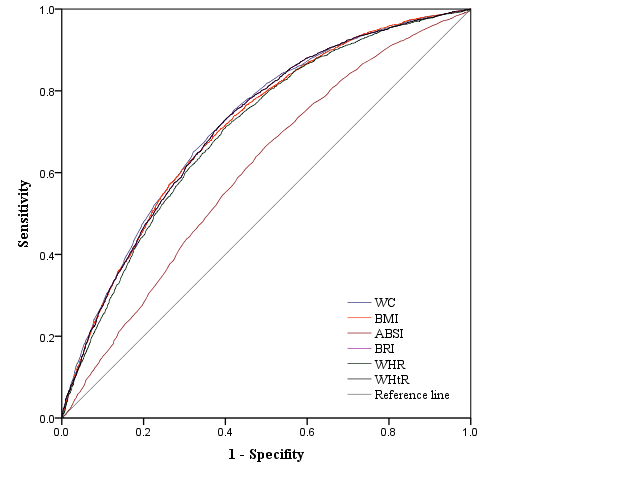

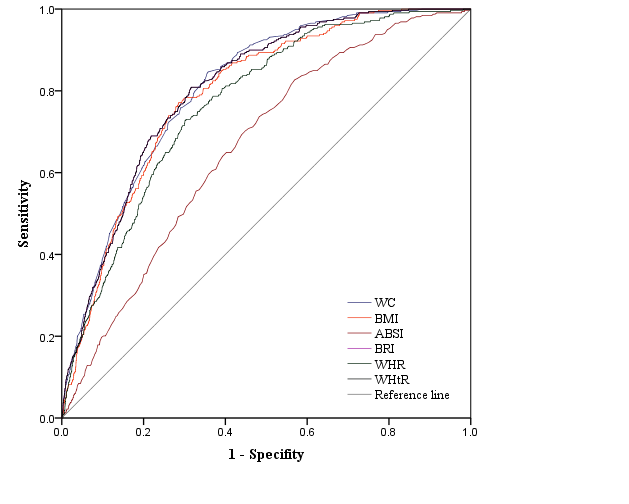


K L


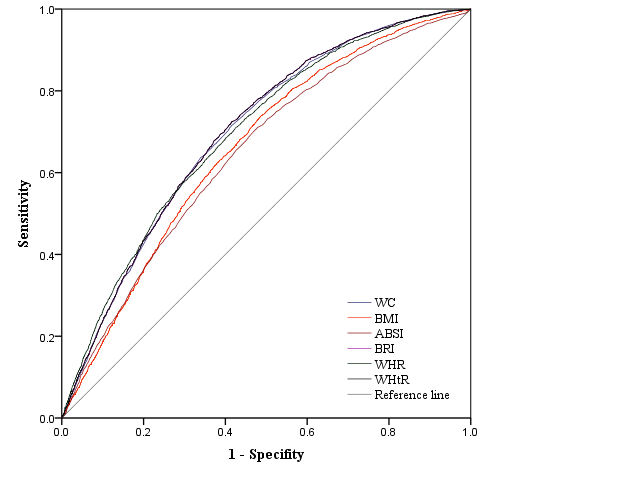

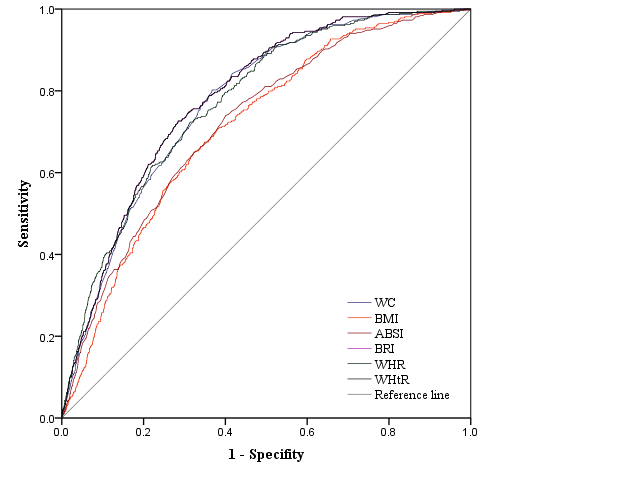


**Figure S1** **Receiver Operating Characteristic Curves (ROCs) for anthropometric indices and serum lipid levels.** (A) ROCs for anthropometric indices and TG level in men, (B) ROCs for anthropometric indices and TC level in men, (C) ROCs for anthropometric indices and LDL-C level in men, (D) ROCs for anthropometric indices and HDL-C level in men, (E) ROCs for anthropometric indices and TG level in women, (F) ROCs for anthropometric indices and TC level in women, (G) ROCs for anthropometric indices and LDL-C level in women, (H) ROCs for anthropometric indices and HDL-C level in women, (I) ROCs for anthropometric indices and one/two categories of abnormal serum lipid indices in men, (J) ROCs for anthropometric indices and three/more categories of abnormal serum lipid indices in men, (K) ROCs for anthropometric indices and one/two categories of abnormal serum lipid indices in women, (L) ROCs for anthropometric indices and three/more categories of abnormal serum lipid indices in women.
